# Medical Students

**Published:** 1836

**Authors:** 


### Art. III.—Medical Students.

In the Boston Medical and Surgical Journal, of June 15th, we have an excellent article from the pen of the editor on Medical Students, which we shall lay before our readers. It seems to us that every Institution, which has for its object the good of the community, should seek to elevate the standard of Medical Education, and thus contribute not only to protect the ignorant but to preserve the dignity of a noble profession. The illiterate and dissipated should not be allowed the honors of any of our Colleges, and it seems to us that the time is not far distant when those which confer degrees upon such will sink into merited disgrace.

It requires talents, integrity of character, patience and persevering industry, to excel in any profession, but in the medical they are all essential, even to a common practitioner. We are aware that with them a well disciplined mind will accomplish much, even when deprived of the advantages of an early education. Still this should by no means be neglected. Indeed, Medical Colleges should require every student to furnish a diploma with the evidences of a good moral character, from a respectable literary institution, or subject him to an examination before he is allowed to matriculate. If this course were adopted, the standard of our profession would be elevated, and many that are now let loose upon society, only to destroy its members, would be returned to the plough, the hammer, or the scenes of dissipation, which they have just left. Medical Institutions certainly owe something to the communities in which they are placed. In the present rage for doctor-making, the principal object with them appears to be *number* rather than *quality* of pupils. A spirit of competition is abroad, in this respect, which is equally as disgraceful to the schools as it is injurious to the profession and the public. But here is the article we referred to.

"Of the multitude of young gentlemen engaged in the study of medicine in the United States, there are doubtless some destined to rise to enviable distinction, whose beginning has been of the most unpromising character; and others, with every possible advantage, who will never be recognized as ornaments of the profession. That there is a natural tact manifested in certain individuals, for conducting the business of a general practitioner, cannot be questioned. One man will acquire reputation, with the desirable accompaniement of wealth, and through a long and active life maintain an elevated standing in society, and yet fall far below the sterling qualifications of a neighbor, who may, perhaps, have struggled with a series of disappointments and vexations, in attempting to gain his daily bread. It has been remarked, that those students who have pursued their studies under the greatest disadvantages, and acquired their knowledge at the expense of all those pleasures which fascinate, distract, and oftentimes corrupt the wealthier class of pupils, usually succeed best, and ultimately triumph over all the difficulties which opposed their progress in the pursuits of science. This proves nothing more, however, than that man, under certain circumstances, doubles his diligence and systematizes his time, and therefore overcomes, by steady perseverance, obstacles which, though apparently insurmountable, only required prudence and economy to subdue. But of all the sustaining powers which a medical student has at his requisition, there is nothing more important to his future success, than a disciplined mind. Next, his habits—those every-day exhibitions which one makes of himself to the argus eyes of the world—are of immense consequence.

At the period when students usually commence the study of medicine, the mind is developed, and those leading traits of character on which their future respectability in life is to depend, show themselves prominently. It involves no spirit of prophecy to predict, with considerable certainty, even in the very beginning, the success with which an individual will pioneer himself into public favor and esteem; —or to mark out the chart of his disappointments, when he swerves from those principles on which all degrees of personal happiness have a positive foundation.

In this age of excitement, medical students are quite as likely to be operated upon by the noxious influences of bad example as any others: indeed under certain circumstances, more so. Some of the collateral departments of medicine seem to expose them to peculiar temptations. Temperance, sobriety of deportment and uncompromising integrity, never failed of sustaining a man in any honorable vocation, in the estimation of those whose favor was worth possessing. Though all cannot become eminent, there is no possible apology for not being strictly upright and virtuous. All deviations from this plain and praiseworthy course are invariably attended with self-reproach, parental grief, and the marked displeasure of all who were disposed to be true friends.

A medical student in our cities is not required by any laws of good society to maintain a race-horse, to devote hours to the theatre which should be exclusively employed in study ; nor is he the less honorable for not wasting in the cup what would conduct him triumphantly through his pupilage. It were to be wished that dissipations of all kinds were frowned upon so severely by the faculties of medical institutions, that no one, known to be habitually intemperate, could be admitted to a degree.

These reflections were elicited, a little time since, in consequence of witnessing, in a barber's shop, the sickening spectacle of a medical student so thoroughly drunk, that it was even difficult for him to articulate his own name. This was not all,—he had fallen on the sharp edge of a granite door-step, and cut a fearful-looking gash under the margin of the lower jaw. Yet this man is called highly respectable, belongs to a fond and indulgent family—doomed, beyond a question, to deplore, hereafter, with aching hearts, the degradation of a child of many prayers. But in this instance there is scarcely a hope of reformation, because no one presumes to tell the gentleman of his faults, and colleges were never known to frown with terrible vengeance on this kind of folly in youth. One who attempted to prove that this same hopeless candidate for honor was guilty of no very reprehensible practice, though he admitted it was unfortunate, felt persuaded that getting intoxicated occasionally would not essentially interfere with any duties he might be called upon to perform. "Why," said he, "the habit is formed, and he must have it." Within a month, in one of his Bacchanalian orgies, an acquaintance took the liberty of taking six hundred dollars from his pockets, which would otherwise have been the spoil of thieves before he became sober. With this shameful vice, which he has not the decency to try to conceal, this same young man is looking forward with delight to his lawful emancipation from books—to do what?—to practice medicine! Ye destinies,—spare the patients of such a physician!

Hereafter, the saying that was once common in this country, that this man and that man are excellent physicians or surgeons, as the case may be, if called when they are sober, will not be heard. Those who indulge any expectations in the profession will be compelled, from necessity, if not by higher motives, to be gentlemen in conduct. A dissipated student ordinarily ends his career as wretchedly as those in humbler walks ; while his moral guilt is vastly greater, from the circumstance that he knew the moral and physical danger, but disregarded the consequences of both.

As the members of the profession are multiplying with rapidity, no second-rate acquirements will suffice. People know readily how to discriminate the qualities most desirable in a physician. To real skill, must be superadded those sterling qualities of character which command the homage of such as value those who appear properly to respect themselves.

Earnestly desiring that these admonitory observations may fall under the eye of him who elicited them, and excite him to an effort to disenthral himself from the iron grasp of a fiend that devours and spares not, we cannot refrain from whispering to any others verging towards the same fearful precipice, to reflect, if but for a moment—'*honor and shame from no condition rise.*'"

The case here given is by no means an uncommon one. Students frequently find their way into cities, for the purpose of attending medical lectures, who spend all their money at card tables or coffee-houses, and at length leave them in disgrace, or borrow from their sober associates, without the most remote idea of ever paying. We have a case of this kind in our eye at present. A young man came to our city, from a distance, highly recommended by relatives in the profession, known to be of the highest standing both as writers and practitioners. He spent his money at the race course or gambling table, while he procured his tickets on credit, and borrowed money to pay his current expenses. At length he was arrested by an officer, and his parent, who had supplied him liberally, refused to furnish him with any more funds. Had this youth, who was highly respectable, attended to his books, instead of scenes of dissipation, he might have been the pride of his friends and an ormanent to the profession, instead of a disgrace to both. Should he still find his way into the community as a doctor, with his present habits, his patients must *feel* the effects of his vices. W. W.

